# Low Dietary Variety Is Associated with Incident Frailty in Older Adults during the Coronavirus Disease 2019 Pandemic: A Prospective Cohort Study in Japan

**DOI:** 10.3390/nu15051145

**Published:** 2023-02-24

**Authors:** Miyuki Yokoro, Naoto Otaki, Megumu Yano, Tomomi Imamura, Norikazu Tanino, Keisuke Fukuo

**Affiliations:** 1 Department of Dietary Life and Food Sciences, Junior College Division, Mukogawa Women’s University, Nishinomiya 663-8558, Hyogo, Japan; 2 Research Institute for Nutrition Sciences, Mukogawa Women’s University, 6-46 Ikebiraki-cho, Nishinomiya 663-8558, Hyogo, Japan; 3 Department of Food Sciences and Nutrition, School of Food Sciences and Nutrition, Mukogawa Women’s University, Nishinomiya 663-8558, Hyogo, Japan; 4 Department of Innovative Food Sciences, School of Food Sciences and Nutrition, Mukogawa Women’s University, Nishinomiya 663-8558, Hyogo, Japan

**Keywords:** SARS-CoV-2 outbreak, frailty, diet, dietary behavior, older adults

## Abstract

Background: Stagnation of social activity due to the COVID-19 pandemic probably reduces motivation to maintain a healthy diet. It is important to report on the dietary changes observed in older adults during a period of restriction on outings and to clarify the relationship between dietary variety and frailty. This one-year follow-up study examined the association between frailty and dietary variety during the COVID-19 pandemic. Methods: Baseline and follow-up surveys were conducted in August 2020 and August 2021, respectively. The follow-up survey was distributed by mail to 1635 community-dwelling older adults aged ≥65 years. Of the 1235 respondents, 1008 respondents who were non-frail at baseline are included in this study. Dietary variety was examined using a dietary variety score developed for older adults. Frailty was assessed using a five-item frailty screening tool. The outcome was frailty incidence. Results: In our sample, 108 subjects developed frailty. A linear regression analysis revealed a significant association between dietary variety score and frailty score (β, −0.032; 95% CI, −0.064 to −0.001; *p* = 0.046). This association was also significant in Model 1, adjusted for sex and age, (β, −0.051; 95% CI, −0.083 to −0.019; *p* = 0.002) and in a multivariate analysis that added adjustments for living alone, smoking, alcohol use, BMI, and existing conditions to Model 1 (β, −0.045; 95% CI, −0.078 to −0.012; *p* = 0.015). Conclusions: A low dietary variety score was associated with an increased frailty score during the COVID-19 pandemic. The restricted daily routine caused by the COVID-19 pandemic will probably continue to have a long-term effect in terms of reduced dietary variety. Thus, vulnerable populations, such as older adults, might require dietary support.

## 1. Introduction

Since first detected in 2019, coronavirus disease (COVID-19) has rapidly impacted the global population, with over 700 million people becoming infected and suffering from severe acute respiratory syndrome [1].

Japan declared a state of emergency in April 2020 when no treatment or vaccine for COVID-19 was available to prevent the spread of infection. During this state of emergency, people were requested to work from home and use online services. Measures were also taken to reduce the opening hours of grocery stores and other businesses [2].

Japan launched its vaccination campaign and began vaccinating against COVID-19 on 17 February 2021. At present, approximately 80% of the population aged ≥12 years have received the required number of vaccinations; the vaccination rate is reported to be over 90% among older adults aged ≥65 years [3,4]. As the campaign proceeded and vaccination rates increased, social restrictions gradually eased. For example, recreation facilities that attract large numbers of people reopened with shorter hours of operation and more restrictions for admission [5]. Older adults and those with a history of diseases are at high risk of COVID-19; therefore, few community activities intended for older adults exist because sufficient space cannot be provided [6,7,8,9,10].

Increased life expectancy is accelerating the aging population proportion worldwide. People aged ≥65 years constituted 9.3% of the world’s population in 2020, and this proportion is predicted to be 17.8% by 2060 [11]. Frailty is significantly common among the elderly, and is characterized by pronounced fragility due to declining physical function. Adverse outcomes such as death, falls, institutionalization, and disability are associated with frailty [12,13,14,15]. An estimated 12% of people aged ≥50 years live with frailty worldwide, and an estimated 8% of people aged ≥65 years live with frailty in Japan [16,17]. Additionally, frailty is associated with an increased risk of serious COVID-19 [18,19,20,21]. For these reasons, identifying factors that prevent frailty is of considerable interest to many countries with aging societies.

Many published studies describe changes in dietary behavior caused by the COVID-19 pandemic, and there are concerns that prolonged deterioration of dietary behavior due to the COVID-19 pandemic reduces disability-adjusted life years [22,23,24,25,26,27,28]. However, we are not aware of any studies that have examined long-term effects of COVID-19 on dietary behavior.

Maintaining a healthy diet prevents frailty. Healthy dietary patterns such as the Mediterranean diet and the Dietary Approaches to Stop Hypertension diet, promote the consumption of a variety of foods that are beneficial to overall health, including the prevention of frailty [29,30]. Dietary variety, which is an important element of a healthy diet, refers to the intake of various food groups during a specific period, not to the amount of food consumed [31]. Healthy dietary behavior improves nutrient adequacy [32]. A higher dietary variety score is associated with faster walking speed and the prevention of a decline in grip strength [33,34,35]. Thus, it is important to document dietary changes observed in older adults whose mobility is restricted, and to clarify the relationship between a diverse diet and frailty.

This study was a one-year follow-up survey in community-dwelling older adults that examined the association between frailty and dietary variety during the COVID-19 pandemic.

## 2. Methods

### 2.1. Study Subjects and Study Period

This prospective cohort study was conducted in Japan in August 2020. We randomly selected 4996 community-dwelling adults from the elderly population aged ≥65 years as prospective study subjects using addresses recorded in the Health and Welfare Department office. Individuals who were hospitalized or who resided in a nursing home were excluded.

The baseline survey forms were distributed by mail in August 2020, at which time subjects were also asked to cooperate in a follow-up survey.

**Figure 1:**
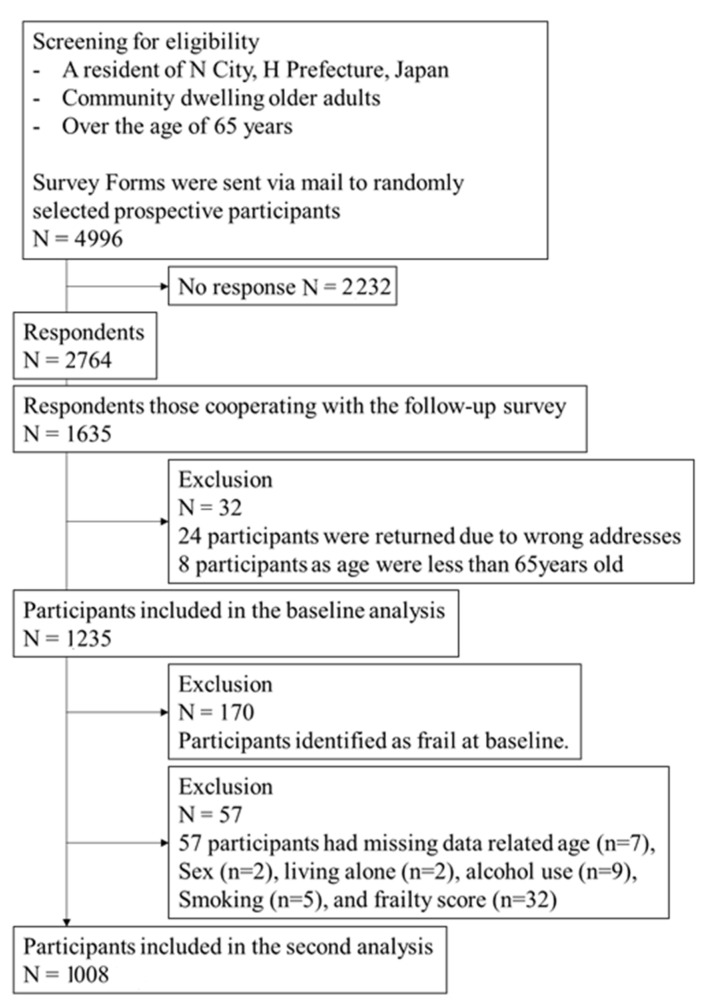
Flowchart for the recruitment of study subjects.

The follow-up survey forms were mailed in August 2021, and 1635 subjects responded. Figure 1 shows the flow chart of this study.

### 2.2. Ethical Approval

Study details were explained in writing to the subjects, and the return of a completed survey form was considered as informed consent for participation. The Ethics Committee of Mukogawa Women’s University approved this study (Approval Number: 20-53).

### 2.3. Survey Content

The survey included demographic questions such as sex, height, weight, age, smoking habits (smoking or non-smoking), drinking habits (drinking alcohol once per week or more or not drinking), and living arrangements (living alone or living with others). Body mass index (BMI) was calculated from the subjects’ self-reported weights and heights. Chronic conditions such as hypertension, diabetes, hyperlipidemia, stroke, and cardiac disease were also self-reported. Social activity was determined by the frequency of interactions with family and friends, as well as the frequency of participation in community activities. The negative impact of COVID-19 on social interactions was assessed by a modified version of the following question from the SF-36: “During the past four weeks, to what extent has your physical health or emotional problems interfered with your normal social activities with family, friends, neighbors, or groups?” [36] Potential responses to this question were “has not hindered at all”, “has hindered very little”, “has hindered somewhat”, “has hindered quite a bit”, “Extremely”, “could not do social activities”, and “No participation in social activities or No separated family, relatives or friends”.

### 2.4. Dietary Variety Score

Subjects completed a food-group-based dietary questionnaire to determine their dietary variety score [37]. This score was calculated by quantifying how frequently an individual consumed foods from across 10 categories: meats, fish and shellfish, eggs and egg products, soybeans and soybean products, milk and milk products, seaweeds, vegetables, fruits, potatoes, and oils. The total score (food) ranged from 0 to 10 points, with the intake of each food group assigned 1 point for a response of “eat almost every day” and 0 for “eat once every two days/eat once or twice a week/eat hardly ever.”

A more varied diet can reduce the risk of high-level functional decline, and can also help maintain physical performance as measured by grip strength and usual gait speed [34,37]. To best represent a long-term diet during a 1-year follow-up period and to account for changes in food consumption, we determined the cumulative mean dietary variety score from two food-group-based dietary questionnaires conducted at baseline and at the 1-year follow-up [38]. A total of 36 Scores of ≤3 points, ≥3 and <6 points, and ≥6 points indicated low, mid, and high dietary variety, respectively.

### 2.5. Frailty Score

The frailty score was calculated from a “yes” or “no” response to the following five questions: “Have you lost 2 kg or more in the past 6 months?”, “Do you think you walk slower than before?”, “Do you go for a walk for your health at least once a week?”, “Can you recall what happened 5 min ago?” and “In the past 2 weeks, have you felt tired without reason?”. The three questions, “Have you lost 2 kg or more in the past 6 months?”, “Do you think you walk slower than before?” and “In the past 2 weeks, have you felt tired without reason?” were assigned a score of 1 point for “yes” and 0 points for “no.” The questions, “Do you go for a walk for your health at least once a week?” and “Can you recall what happened 5 min ago?” were scored as 1 point for “no” and 0 points for “yes.” The frailty score was the total score for all five questions, and could range from 0 to 5 points [37]. Scores of ≤2 and ≥3 points indicated non-frail and frail status, respectively.

Based on the frailty score, frail older adults had significant risk of care insurance use after two years. The self-report questionnaire for frailty has predictive validity for disability in older Japanese adults [39].

### 2.6. Statistical Analysis

Statistical analyses were performed using IBM SPSS 25.0. Categorical data are displayed as number of respondents and percentages, while continuous variables are displayed as mean and standard deviation. To compare subjects exhibiting frailty with those exhibiting non-frailty, the Mann–Whitney U and chi-squared tests assessed the quantitative and categorical variables.

Logistic regression analysis assessed associations between dietary variety score and frailty; Model A was adjusted for sex and age, while Model B included adjustments for BMI, alcohol use, smoking, living alone, self-reported hypertension, diabetes, hyperlipidemia, stroke, and cardiac disease. Additionally, linear regression was used to examine associations between the dietary variety and frailty scores; Model 1 was adjusted for sex and age, while Model 2 included adjustments for BMI, alcohol use, smoking, living alone, self-reported hypertension, diabetes, hyperlipidemia, stroke, and cardiac disease. Statistical significance was defined as a two-tailed *p* value of <0.05.

## 3. Results

Of the 2764 original subjects, 1235 responded to the one-year follow-up survey; 170 subjects who were frail at baseline were excluded from the analysis. Finally, 1008 subjects were eligible after exclusion of those with missing data related to sex (n = 2), age (n = 7), living alone (n = 2), alcohol use (n = 9), smoking (n = 5), and frailty score (n = 32). A flow chart describing the selection process of this study sample is shown in Figure 1. Of the 1008 subjects, 11.2% (113 subjects) were determined to be frail after one year. The incidence was 112.1 cases per 1000 person-years.

Table 1 shows the potential confounders according to baseline characteristics such as age, sex, body mass index, alcohol intake, smoking status, history of disorders [40,41]. and compares the basic characteristics of subjects with frailty and non-frailty. The mean of the two dietary variety scores was significantly lower in subjects with frailty than in subjects with non-frailty. No significant difference was found between the proportion of male subjects with frailty and those with non-frailty. The mean age of subjects with frailty (75.7 years) was significantly higher than that those with non-frailty (73.8 years) (*p* = 0.002). DVS was significantly higher (*p* = 0.048) in subjects with non-frailty than in subjects with frailty.

The change in consumption of various food groups over time is listed in Table 2. The frequency of milk and dairy product intake in subjects with non-frailty showed a decreasing trend (*p* = 0.076), as did the intake of seaweed (*p* = 0.054). The frequency of intake of meats decreased significantly in subjects with frailty (*p* = 0.031), and the frequency of intake of soybeans and soybean products also showed a decreasing trend (*p* = 0.054). The frequency of intake of eggs showed an increasing trend (*p* = 0.078). A significant decrease was observed in the frequency of intake of milk and dairy products (*p* = 0.032) in all subjects.

Linear regression analysis revealed a significant association between dietary variety score and frailty score (β, −0.032; 95% CI, −0.064 to −0.001; *p* = 0.046; Table 3). This association was also significant in Model 1 adjusted for sex and age (β, −0.051; 95% CI, −0.083 to −0.019; *p* = 0.002) and in a multivariate analysis that added adjustments for living alone, smoking, alcohol use, BMI, and existing conditions to Model 1 (β, −0.045; 95% CI, −0.078 to −0.012; *p* = 0.015). In a sensitivity analysis excluding subjects with low dietary variety scores less than 1 (n = 21), the association between frailty score and dietary variety score remained significant in the multivariate models (β,−0.047; 95% CI, −0.081 to −0.013; *p* = 0.008)

Table 4 shows the subjects characteristics based on food variety score. The variety score level were significantly associated with gender, BMI, alcohol intake, smoking status and higher prevalence of hypertension.

Table 5 shows the association between dietary variety score and incident frailty among subjects with non-frailty at baseline. Compared to that among subjects with a high dietary variety score, the odds ratio (OR) for the onset of frailty among subjects with a low dietary variety score was 1.648 (95% confidence interval [CI], 0.941–2.887; *p* = 0.081). This association was significant in Model A, which adjusted for sex and age, (OR, 1.911; 95% CI, 1.066–3.426; *p* = 0.030) and in a multivariate analysis that added adjustments for living alone, smoking, alcohol use, BMI, and existing conditions to Model A (OR, 1.877; 95% CI, 1.034–3.409; *p* = 0.039).

The effect of the COVID-19 pandemic on social activity during the surveyed period is noted in Table 6. The COVID-19 pandemic hindered participation in social activities and meeting with family and friends among at least half the community-dwelling older adults. No significant difference was seen in the frequency of interaction with family and friends during the pandemic. However, several subjects had less frequent interaction and contact with their friends and family.

## 4. Discussion

This study investigated the change in dietary variety and frailty score among community-dwelling older adults over a one-year period during the COVID-19 pandemic. The dietary variety score during the surveyed period was significantly lower in subjects with frailty than in subjects with non-frailty. This study revealed the change in dietary variety during the survey period. Furthermore, a lower dietary variety score during the one-year period was positively associated with frailty score. This study did not include an assessment of DVS scores before the COVID-19 pandemic. Therefore, it is unclear whether the pandemic worsened DVS scores. However, at a minimum, the study shows that low DVS scores over one year of the COVID-19 pandemic are associated with an increased risk of frailty.

The incidence rate of frailty in Japan is reported to be 8.7% [17]. A meta-analysis reported a 13.6% incidence rate of frailty, or 43.4 cases/1000 person-years, among older adults with non-frailty during a median 3-year follow-up period [40]. In this study, the incidence rate of new frailty cases during the one-year follow-up period was 11.2% or 112.1 cases/1000 person-years, which is higher than the incidence rates reported in previous studies [17,40]. In this study, frailty was assessed in a self-administered format. Furthermore, the COVID-19 pandemic may cause subjects to be overly negative when evaluating their health. These factors may have caused the higher incidence rate of frailty in this study compared to those in previous reports.

Several reports have shown changes in dietary behavior during the COVID-19 pandemic; however, almost all have examined the beginning of the pandemic [22,23,24,25,26,27]. Some of these reports have noted that a deterioration in diet due to the pandemic is negatively associated with frailty, functional limitations, and undernutrition [42,43,44,45,46]. In the early part of the pandemic, the restricted access to food caused by measures that reduced the opening hours of groceries and other businesses may have reduced the quality of people’s meals [43,44]. This decline in the quality of food caused by restricted access to food is probably a short-term effect. Restricted daily activities have continued for approximately 18 months due to the pandemic. This study did not assess dietary variety before the pandemic; thus, it cannot identify the changes in dietary variety at the beginning of the pandemic. However, the restricted daily routine caused by the COVID-19 pandemic will probably have a long-term effect in terms of reduced dietary variety.

This study revealed that social interaction between community-dwelling older adults and others is greatly limited by the COVID-19 pandemic. Due to the pandemic, restrictions on movement were in place in Japan for almost the entire period from February 2021 to August 2022. The campaign for vaccination is also significantly active in Japan, where the proportion of older adults aged 65 years or older with two or more vaccinations is over 90% [3,4]. Nonetheless, organizing community activities for older adults is difficult. This is because sufficient indoor space cannot be provided, and older adults and those with a history of diseases are at high risk of severe symptoms and death [7,8,9,10].

Social activity is a key factor in maintaining not a healthy diet but healthy life-style [47,48,49,50,51,52,53]. Stagnation of social activity due to the pandemic probably reduces motivation to maintain a healthy diet. In a study by Conklin et al., a lower level of contact with friends was associated with the reduced consumption of a wide variety of fruits and vegetables [53] The stagnation of social activities among older adults due to the pandemic will probably make it difficult for older adults to maintain a healthy diet.

This might be one of the factors that explain the acceleration of frailty due to the COVID-19 pandemic.

This large-scale follow-up survey conducted during the pandemic has some limitations. First, the follow-up survey was completed by a low percentage of subjects. This might have led to a nonresponse bias. The generalizability of findings may be limited. Second, no weighting methods were used in the dietary surveys. A dietary variety score does not evaluate the intake of specific nutrients, and an accurate evaluation of the association between frailty and diet requires an evaluation of the intake of specific nutrients, such as protein. Third, social desirability bias might have been present in responses. The pandemic may cause people to be overly negative when evaluating their own health and dietary situation. This study also evaluated frailty and dietary variety based on self-reporting by subjects. This suggests that associations may be overestimated in this study. Fourth, the surveys were conducted one-year apart, which is a short period of observation. Both surveys in this study fell in the middle of the COVID-19 pandemic. Although the number of new daily COVID-19 infections in Japan fell below 100 in October 2021, the pandemic subsequently spread again and reached over 100,000 daily infections for the first time, in February 2022. The COVID-19 pandemic is expected to persist long-term; thus, further follow-up surveys will be required. Finally, the food variety was not assessed before the pandemic; hence, dietary variety scores cannot be compared before and after the onset of the pandemic. Nevertheless, this study revealed the change in dietary variety during the surveyed one-year period.

## 5. Conclusions

In conclusion, this study involved a one-year follow-up survey of older adults during the COVID-19 pandemic and examined the association between dietary variety and frailty. The responses revealed an association between frailty and dietary variety during the COVID-19 pandemic. The restricted daily routine caused by the COVID-19 pandemic will probably have a long-term effect in terms of reduced dietary variety. Thus, vulnerable populations, such as older adults, might require dietary support.

## Figures and Tables

**Table 1:** Baseline characteristics.

		All Subject n = 1008		Non-Frail Subject n = 895		Frailty Subject ^+^ n = 113		*p* Value
		n or Mean	Percent or SD	n or Mean	Percent or SD	n or Mean	Percent or SD	
Sex	Men	476	47.2	421	47.0	55	48.7	0.829
	Female	532	52.8	474	53.0	58	51.3	
Age	(years)	73.8	(5.7)	73.6	(5.7)	75.4	(6.0)	0.002
Height	(m)	1.60	(0.08)	1.60	(0.08)	1.60	(0.08)	0.771
Body weight	(kg)	58.2	(10.4)	58.0	(10.3)	59.0	(11.3)	0.364
Body mass index	(kg/m^2^)	22.5	(2.9)	22.5	(2.9)	22.9	(3.0)	0.244
Living alone	Others	815	80.9	719	80.3	96	85.0	0.257
	Living alone	193	19.1	176	19.7	17	15.0	
Alcohol intake	Not drinking	491	48.8	429	47.9	62	54.9	0.327
	Drinking	517	51.2	466	52.1	51	45.1	
Smoking status	Non-smokers	625	62.0	558	62.3	67	59.3	0.821
	Past smokers	318	31.5	280	31.3	38	33.6	
	Current Smokers	65	6.4	57	6.4	8	7.1	
History of disorders	Cancer	53	5.3	45	5.0	8	7.1	0.368
	Cardiovascular diseases	110	10.9	94	10.5	16	14.2	0.261
	Cerebrovascular diseases	14	1.4	10	1.1	4	3.5	0.062
	Hypertension	356	35.3	314	35.1	42	37.2	0.677
	Diabetes	125	12.4	102	11.4	23	20.4	0.010
	Hyperlipidemia	112	11.1	107	12.0	5	4.4	0.016
Dietary Variety Score		4.35	1.93	4.39	1.94	4.01	1.86	0.048
Frailty score	baseline	1.05	0.77	0.99	0.77	1.57	0.57	Time × group
	One-year follow-up	1.04	0.77	1.03	0.77	3.12	0.32	*p* < 0.001

^+^ Subjects with frailty: Frailty was defined as the status when Frailty score is 3 points or more.

**Table 2:** Changes in dietary variety during one-year follow-up survey *.

		All Subjects					Non-Frailty Subjects					Frailty Subjects ^+^				
		n = 1008					n = 895					n = 113				
		Baseline		One-Year After		*p* Value *	Baseline		One-Year After		*p* Value	Baseline		One-Year After		*p* Value
		n	(%)	n	(%)		n	(%)	n	(%)		n	(%)	n	(%)	
Meat	Less than 3 times per week	577	57.2	586	58.1	0.616	519	58.0	516	57.7	0.895	58	51.3	70	61.9	0.031
	Everyday	431	42.8	422	41.9		376	42.0	379	42.8		55	48.7	43	38.1	
Fish	Less than 3 times per week	825	81.8	840	83.3	0.258	733	81.9	743	83.0	0.440	92	81.4	97	85.8	0.332
	Everyday	183	18.2	168	16.7		162	18.1	152	17.0		21	18.6	16	14.2	
Egg	Less than 3 times per week	517	51.3	501	49.7	0.314	444	49.6	437	48.8	0.703	73	64.6	64	56.6	0.078
	Everyday	491	48.7	507	50.3		451	50.4	458	51.2		40	35.4	49	43.4	
Soy and Soy products	Less than 3 times per week	535	53.1	560	55.6	0.119	470	52.5	484	54.1	0.370	65	57.5	76	67.3	0.054
	Everyday	473	46.9	448	44.4		425	47.5	411	45.9		48	42.5	37	32.7	
Milk and daily products	Less than 3 times per week	215	21.3	242	24.0	0.032	189	21.1	210	23.5	0.076	26	23.0	32	28.3	0.263
	Everyday	793	78.7	766	76.0		706	78.9	685	76.5		87	77.0	81	71.7	
Seaweeds	Less than 3 times per week	838	83.1	856	84.9	0.086	744	83.1	763	85.3	0.054	94	83.2	93	82.3	1.000
	Everyday	170	16.9	152	15.1		151	16.9	132	14.7		19	16.8	20	17.7	
Colored vegetables	Less than 3 times per week	329	32.6	339	33.6	0.536	282	31.5	297	33.2	0.306	47	41.6	42	37.2	0.424
	Everyday	616	67.4	669	66.4		613	68.5	598	66.8		66	58.4	71	62.8	
Fruits	Less than 3 times per week	392	38.9	384	38.1	656	346	38.7	334	37.3	0.425	46	40.7	50	44.2	0.541
	Everyday	616	61.1	624	61.9		549	61.3	561	62.7		67	59.3	63	55.8	
Potatoes	Less than 3 times per week	908	90.1	918	91.1	0.395	805	89.9	814	90.9	0.426	103	91.2	104	92.0	1.000
	Everyday	100	9.9	90	8.9		90	10.1	81	9.1		10	8.8	9	8.0	
Oils	Less than 3 times per week	538	53.4	558	55.4	0.182	475	53.1	496	55.4	0.136	63	55.8	62	54.9	1.000
	Everyday	470	46.6	450	44.6		420	46.9	399	44.6		50	44.2	51	45.1	

* Statistical analysis was performed to compare the baseline and one-year follow-up period in each group. ^+^ Subjects with frailty: Frailty was defined as the status when the Frailty score is 3 points or more.

**Table 3:** Linear regression analysis between frailty score and dietary variety score during one-year follow-up of non-frail subject at baseline.

		B	95% CI		*p* Value
			Lower	Upper	
Men					
	Crude	−0.047	−0.093	−0.002	0.042
	Model 1a	−0.068	−0.114	−0.023	0.003
	Model 2b	−0.059	−0.104	−0.013	0.012
Female					
	Crude	−0.022	−0.068	0.023	0.338
	Model 1a	−0.034	−0.080	0.012	0.143
	Model 2b	−0.027	−0.075	0.020	0.258

Model 1a: adjusted for age. Model 2b: model 1 plus further adjustment for BMI, alcohol use, smoking, living alone, self-reported hypertension, diabetes, hyperlipidemia, stroke, cardiac disease and cancer.

**Table 4:** Subject characteristics based on food variety score.

		High, 6 Points or More		Mid, 3 Points or More and Less than 6 Points		Low, Less than 3 Points		*p*_trend_
		n or Mean	Percent or SD	n or Mean	Percent or SD	n or Mean	Percent or SD	
Sex	Men	80	33.1	213	44.6	183	63.5	*p* < 0.001
	Female	162	66.9	265	55.4	105	36.5	
Age	(years)	74.5	(6.0)	74.2	(5.7)	72.6	(5.4)	*p* < 0.001
Height	(m)	158.5	(8.0)	159.9	(8.6)	162.3	(8.2)	*p* < 0.001
Body weight	(kg)	54.8	(9.6)	57.9	(9.8)	61.4	(11.0)	*p* < 0.001
Body mass index	(kg/m^2^)	21.7	(2.7)	22.5	(2.7)	23.2	(3.3)	*p* < 0.001
Living alone	Others	193	79.8	384	80.3	238	82.6	0.388
	Living alone	49	20.2	94	19.7	50	17.4	
Alcohol intake	Not drinking	129	53.3	241	50.4	121	42.0	*p* < 0.001
	Drinking	113	46.7	237	49.6	167	58.0	
Smoking status	Non-smokers	182	75.2	308	64.4	135	46.9	*p* < 0.001
	Past smokers	52	21.5	150	31.4	116	40.3	
	Current Smokers	8	3.3	20	4.2	37	12.8	
History of disorders	Cancer	10.0	4.1	27.0	5.6	16.0	5.6	0.485
	Cardiovascular diseases	21	8.7	49	10.3	40	13.9	0.051
	Cerebrovascular diseases	2	0.8	7	1.5	5	1.7	0.380
	Hypertension	56	23.1	189	39.5	111	38.5	*p* < 0.001
	Diabetes	22	9.1	70	14.6	33	11.5	0.485
	Hyperlipidemia	32	13.2	52	10.9	28	9.7	0.207
Frailty score	One-year follow-up	1.2	(1.0)	1.3	(1.0)	1.4	(1.0)	0.036

**Table 5:** Odds ratio for frailty during one-year follow-up of subjects with non-frailty at baseline.

	High, 6 Points or More	Mid, 3 Points or More and Less than 6 Points				Low, Less than 3 Points			
	Ref	OR	95% CI		*p* Value	OR	95% CI		*p* Value
			Lower	Upper			Lower	Upper	
Case subjects/subjects (%)	21/221 (8.7%)	53/478 (11.1%)				31/249 (13.5%)			
Crude Odds ratio	1.000	1.312	0.772	2.232	0.316	1.648	0.941	2.887	0.081
Model A	1.000	1.355	0.793	2.316	0.267	1.911	1.066	3.426	0.030
Model B	1.000	1.294	0.749	2.236	0.356	1.877	1.034	3.409	0.039

Model A: adjusted for sex and age; Model B: model A plus further adjustment for BMI, alcohol use, smoking, living alone, self-reported hypertension, diabetes, hyperlipidemia, stroke, cardiac disease, and cancer.

**Table 6:** Impact of COVID-19 pandemic on social activity during follow-up period.

		All Subjects		Non-Frailty		Frailty		*p* Value
		n = 1008		n = 895		n = 113		
		n	(%)	n	(%)	n	(%)	
Hindered frequency of participation in social organizations								0.024
	Not at all	40	4.3	40	4.5	40	2.7	
	Very little or somewhat	262	26.0	244	27.3	18	15.9	
	Quite a bit or extremely	394	39.1	350	39.1	44	38.9	
	Could not do social activity	105	10.4	92	10.3	13	11.5	
	No participation in social activities	197	19.5	162	18.1	35	31.0	
	Missing value	7	0.7	7	0.8			
Hindered frequency of interaction								
With family								0.477
	Not at all	84	8.3	73	8.2	11	9.7	
	Very little or somewhat	371	36.8	336	37.5	35	31.0	
	Quite a bit or extremely	532	52.8	469	52.4	63	55.8	
	No separated family or relatives.	20	2.0	16	1.8	4	3.5	
	Missing value	1	0.1	1	0.1			
With friends								0.605
	Not at all	53	5.3	48	5.4	5	4.4	
	Very little or somewhat	308	30.6	275	30.7	33	29.2	
	Quite a bit or extremely	593	58.8	528	59.0	65	57.5	
	No friends	53	5.3	43	4.8	10	4.8	
	Missing value	1	0.1	1				
Hindered frequency of contact								
With family								0.142
	Not at all	503	49.9	459	51.3	44	38.9	
	Very little or somewhat	374	37.1	328	36.6	46	40.7	
	Quite a bit or extremely	109	10.8	91	10.2	18	15.9	
	No separated family or relatives.	21	2.1	17	1.9	4	3.5	
	Missing value	1	0.1			1	0.9	
With friends								0.357
	Not at all	349	34.6	316	35.3	33	29.2	
	Very little or somewhat	406	40.3	359	40.1	47	41.6	
	Quite a bit or extremely	200	19.8	176	19.7	24	21.2	
	No friends	51	5.1	42	4.7	9	8.0	
	Missing value	2	0.2	2	0.2			

## Data Availability

The data that support the findings of this study are not publicly available due to their containing information that could compromise the privacy of the research subjects. The data are available from the corresponding author upon reasonable request.
